# Schooling and intimate partner violence: retrospective analysis of India’s Sarva Shiksha Abhiyan using quasi-experimental techniques

**DOI:** 10.1136/bmjph-2024-001530

**Published:** 2025-07-15

**Authors:** Arindam Nandi, Nicole Haberland, Meredith Kozak, Fatima Zahra, Thoai D Ngo

**Affiliations:** 1 Population Council, New York, New York, USA; 2 One Health Trust, Washington, DC, USA; 3 Heilbrunn Department of Population and Family Health, Columbia University, New York, New York, USA

**Keywords:** Community Health, Public Health, Education, education

## Abstract

**Background:**

Almost one-third of ever-married women in India experience physical, psychological or sexual violence by their husbands or partners. In this study, we examined the associations of universal primary education with long-term intimate partner violence (IPV) rates and attitudes condoning IPV among women in India.

**Methods:**

We used data from the National Family Health Survey 2019–2021 and compared women who were eligible for *Sarva Shiksha Abhiyan* (SSA)—a national programme of universal primary schooling implemented in 2001—with women who were older and not eligible for SSA. We employed a quasi-experimental method of propensity score matching and fixed effects regression analyses, accounting for a rich set of background socioeconomic and demographic characteristics as covariates.

**Results:**

Intervention group women who were originally eligible for SSA in 2001 (4 years below the primary to secondary transition age of 14 years) were 16%–31% less likely to justify IPV or experience emotional violence than control group women who were not eligible for the programme (4 years above the age cut-off). There were no statistically significant associations between SSA eligibility and the rates of physical IPV experienced by women. The results were robust to a series of sensitivity analyses and alternate model specifications.

**Conclusions:**

Our findings indicate that universal access to primary schooling may play an important role in reducing IPV and improving gender equality in India and similar low-income and middle-income countries.

**WHAT IS ALREADY KNOWN ON THIS TOPIC**

- Almost one-third of ever-married women in India experience intimate partner violence (IPV), a rate that is among the highest in the world. In addition, almost half of ever-married women in India condone IPV. Previous studies from Turkey, China, Peru, Uganda and other countries in sub-Saharan Africa show that schooling policies (eg, school construction or free and mandatory schooling) may reduce the incidence of IPV or reduce attitudes of IPV acceptability among women. These potential social benefits of education remain inadequately evaluated in the context of India.

**WHAT THIS STUDY ADDS**

- Schooling and female empowerment may reduce IPV, but the relationship may be cyclical as IPV itself may act as a barrier to school enrolment and grade progression. We evaluated the associations between India’s primary schooling expansion programme, known as *Sarva Shiksha Abhiyan* of 2001, and long-term IPV outcomes of women in India. Women who originally benefitted from the programme had improved attitudes towards IPV and lower incidence of emotional IPV experience.

**HOW THIS STUDY MIGHT AFFECT RESEARCH, PRACTICE OR POLICY**

- Our analysis shows that educational policies such as primary schooling expansion may have large secondary public health benefits such as reduction in IPV.

## Introduction

 Intimate partner violence (IPV) against women remains an important public health and social challenge, especially in many low-ncome and middle-income countries (LMICs). In 2019, an estimated 85 000 deaths among women, along with substantial morbidity—equivalent to 8.5 million disability-adjusted life-years lost—were attributed globally to IPV.^1^ Countries in sub-Saharan Africa and South Asia have some of the highest rates of IPV—an estimated 44% and 35% of ever-married or ever-partnered women aged 15–49 years in central sub-Saharan Africa and South Asia, respectively, experience IPV in their lifetime.^1 2^

India’s rapid economic growth and development in recent decades have not been matched with progress with respect to IPV. The most recent National Health and Family Survey of India 2019–2021 (NFHS-5) shows that the proportion of women experiencing IPV has remained largely unchanged during the past decade. More than 100 million Indian women aged 15–49-years (equivalent to 29% of women in this age group) report experiencing any IPV in their lifetime.^1 3^ In addition to being a serious violation of human rights, such violence can have severe detrimental effects on women’s physical and mental health, leading to lower use of family planning methods and higher rates of unwanted pregnancies, abortion, miscarriages, cardiovascular diseases, chronic pain and injury, mental distress and suicidal tendencies.^4^,^11^ Children who grow up in families where the mothers experience IPV can have delayed cognitive and socioemotional development as compared with others, which likely contributes substantially to the risk of early life developmental failure that some 58 million under-5 children in India experience.^12^,^14^

Reducing IPV against women is a complex socioeconomic and public policy task. A recent Lancet series on violence against girls and women identified several effective interventions for reducing IPV in LMICs, including community-based interventions, participatory advocacy movements and women’s empowerment programmes.^15^ Improved school enrolment, attainment and learning levels could also empower women and reduce the prevalence of IPV and improve attitudes towards IPV—a recent World Bank report estimated that women with some or completed secondary-level education have 11%–36% lower risk of experiencing IPV.^16 17^ However, evaluating the causal effect of schooling on IPV outcomes is difficult due to endogeneity, that is, shared antecedents such as underlying economic, social and systemic conditions. In many societies, high prevalence of IPV and low female schooling may both be driven by regressive social and cultural norms such as the dowry system in South Asia.^18^,^21^ This is also reflected in attitudes towards IPV—an estimated 53% ever-married women and 41% of adult men in South and South East Asia (47% of women and 42% of men in India) justify a husband or partner hitting or beating the wife.^22^

While one additional year of schooling in LMICs has been linked with a 1–9% reduction in IPV prevalence, IPV itself may act as a barrier to enrolling and staying in school for adolescent and young adult girls, thereby creating a cyclical relationship with education.^23^,^30^ Education policy initiatives that are mainly designed to improve schooling and learning outcomes without directly affecting other social outcomes such as IPV may help overcome this analytical challenge. In this study, we examined the potential benefits of India’s national primary schooling expansion programme in reducing long-term IPV prevalence and attitudes towards IPV. In 2001, the government of India implemented *Sarva Shiksha Abhiyan (*SSA, or Universal Education Initiative) with the goal of providing 8 years of primary schooling to all eligible children (6–14 years) for free. SSA invested heavily in educational infrastructure by building new schools, improving existing schools, hiring and training more teachers, and increasing classroom resources. The programme reduced the number of out-of-school children from 32 million in 2001 to 9.4 million in 2005, and it has been linked with improved long-term schooling attainment and literacy in adulthood.^31^,^33^

We used data from a large national household survey and compared IPV attitude and experience outcomes of ever-married adult Indian women who were originally eligible for SSA (aged 14 years or below) as compared with those who were above primary school age (age 15 years and above) in 2001. Systematic differences between the two groups were mitigated by employing propensity score matching (PSM) and fixed effects regression analyses that accounted for a rich set of individual, family, and location characteristics. We tested the robustness of our findings through variations in analytical methods and study duration and reported estimates for all women as well as subsamples of location, caste and standard of living.

## Methods

### Details of SSA

Since the 1990s, the Indian government has invested heavily in improving access to, and the quality of, public schools across the country. The District Primary Education (DPEP) programme of 1994 was the first large scale primary schooling initiative that covered 217 out of 466 districts in India.^34^ DPEP targeted districts with poor educational outcomes and increased their educational budgets by up to 20% annually, building new schools, improving existing schools, providing free textbooks and other resources, and training teachers.^34^,^37^ The programme was supported primarily through external assistance from the World Bank and other agencies, with a total expenditure of US$2 billion through the end of the programme in 2000.^38 39^

Following the success of the regional DPEP programme, India launched a national primary schooling expansion programme called SSA in 2001. The main components of the programme were similar to DPEP, with substantially larger investments in construction and repair of schools, teacher hiring and training, and improving access to quality teaching material. During its first 5 years, SSA built 118 000 new schools, upgraded 221 000 existing schools, appointed 386 000 new teachers and annually trained 2.2 million teachers.^31^ In line with a constitutionally mandated joint responsibility of education service delivery assigned to both the central and state governments in India, expenses for implementing SSA were initially split 85% and 15% between the two levels of government, and later changed to equal contributions.^40^ By 2010, SSA had spent US$28 billion to improve access to quality primary schooling around the country.^38 39^

Previous studies have linked both DPEP and SSA with improvements in schooling enrolment rates by 2%–10% and 0.16 extra schooling years completed among eligible children.^33^,^35^ In addition, a few studies have examined the long-term effects of the programmes. Two studies have linked DPEP with higher earnings for unskilled workers in later life and intergenerational benefits in terms of improved learning outcomes for participating parents’ children.^41 42^ Two other yet unpublished studies used national survey data and estimated that DPEP increased long-term schooling grade attainment of women by up to 1 year and reduced rates of IPV against them by 9%–26%.^43 44^ Finally, in another large national study, we linked SSA with 0.2 higher grade attainment, 0.04 points higher literacy test scores and 0.12 higher weight-for-height z-score in adulthood, with similar estimates for men and women.^32^ However, whether SSA reduced the prevalence of women experiencing IPV or improved their attitudes towards IPV remains underexplored.

### National Family Health Survey (2019–2021) data

We used data from the fifth round of the NFHS-5, of India.^45^ NFHS surveys are large nationally representative health surveys that are conducted approximately once every 4–6 years in India as part of the multicountry Demographic and Health Survey initiative.^46^ NFHS-5 was conducted during 2019–2021, covering 636 699 households and 2.8 million individuals from all 707 districts of India. The primary respondents of the survey were 724 115 ever-married (ie, currently or previously married) women of age 15–49 years. These women responded to a household questionnaire that collected socioeconomic and demographic characteristics of the household and its members, including indicators for location, religion, caste, age, sex, years of schooling completed and employment status. In addition, a health questionnaire was administered to the women that collected detailed data on their sexual and reproductive health, family planning, childcare practices and intrahousehold bargaining power and experience of IPV. Data were collected in-person using a Computer Assisted Personal Interviewing tool, and the response rate for the women’s questionnaire was 97%.^47^

We considered the following four binary indicators of attitudes towards IPV that were available in the ever-married women’s health questionnaire: whether the respondent thinks that beating by a husband or partner is justified if she (1) burns food while cooking, (2) neglects their children, (3) argues with husband or partner or (4) goes out of the house without informing the husband or partner. The responses to these questions were coded as binary responses (yes/no). We also examined two indicators of whether within the 12 months preceding the survey, the woman experienced any: (5) emotional violence by the husband or partner and (6) physical violence by the husband or partner. Emotional violence was defined as feeling humiliated, threatened or insulted by the husband or partner and is measured through a combined binary indicator of experiencing at least one act of emotional violence with responses ‘often’ or ‘sometimes’ coded as 1 and ‘never’ coded as 0.^48^,^51^ Similarly, the binary indicator of physical violence measured whether the woman experienced (often or sometimes) at least one of six acts of violence such as being kicked, slapped or pushed by the husband or partner. All IPV indicators in our analysis were self-reported by women, and the data collection tool was designed based on internationally accepted Revised Conflict Tactics Scales measures of IPV.^48^ Survey enumerators ensured the privacy of the women during the interviews, to avoid interference by their partners or other household members.

We considered women who were of elementary school age in 2001 in the intervention group (SSA exposed, ie, eligible to benefit from the programme) while those who were 15 years or older in the control group (SSA ineligible, since they should be in secondary school). While NFHS-5 did not collect information on whether women in our data enrolled in schools specifically due to SSA, previous studies show that the programme substantially increased school enrolment and attainment rates nationwide.^32^,^35^ Therefore, we examined the associations of SSA with IPV outcomes in a manner similar to ‘intention-to-treat’ (ie, exposure to the programme) analysis, which is commonly done in evaluation studies of large scale programmes that have no available data on beneficiary participation.^3252^,^54^

Considering that all children aged 6–14 years were eligible for elementary schooling (and therefore, SSA) in 2001, the differences in background characteristics of the intervention and control groups may be substantially large due to the large age range. To mitigate such differences and make the two groups comparable, we considered individuals who were just above and below the cut-off age for transition from elementary to secondary level of schooling in 2001. We included women who were 11–14 years old in 2001 in the intervention group and those who were 15–18 years old in the control group. In additional analyses, described later, we considered variations in this 4 year study period before and after SSA.

Our analysis included a rich set of covariates to account for differences in the background characteristics of the intervention and control groups. These covariates were selected based on the previously published evaluation studies that used NFHS data from India. 3241 42 52,57At the individual level, these included: age of the woman in years, squared value of age (to incorporate potential non-linear relationship between age and IPV outcomes) and indicators of the woman’s relationship to the household head (whether self, wife, child, daughter-in-law or grandchild). At the household level, we included the number of household members (to account for resource sharing), indicators of location (whether rural vs urban), caste group (scheduled caste (SC), scheduled tribe (ST) and other backward classes (OBC)) and religion (Muslim, Christian or Sikh). SC, ST and OBC are socioeconomically disadvantaged minority groups, as designated by the Indian government, which accounted for 23%, 10% and 44% of the national population respectively in NFHS-5. Caste categorisation is used for affirmative action programmes and policies of the government. Age and sex (whether female) of the household head were also included. Standard of living of the household was measured using a composite index of ownership of durable assets such as TV, car, bicycle and telephone, along with living condition indicators such as construction quality and the number of rooms of the dwelling unit. Households were grouped into five wealth quintiles based on this index, and we included indicators of the top (richest) four quintiles in our analysis.

### PSM analysis

We used PSM methods to estimate the associations between SSA and IPV outcomes among women. PSM is a widely used quasi-experimental analysis technique for observational settings.^58^,^61^ In our analysis, individuals in the intervention group (SSA exposed) and control group (SSA ineligible) may be different from each other in terms of their socioeconomic and demographic characteristics. If such differences were also correlated with the IPV indicators, least squares regression estimates of the association between SSA and our outcome variables would be biased. For example, women in the control group were on average older than women in the intervention group. If these older women were also less likely to experience IPV and justify IPV than younger women, an estimated negative association between SSA and IPV outcomes would be smaller in magnitude than the true estimate. PSM can reduce such systematic differences by matching each individual in the intervention group with one or more observationally similar individuals in the control group, and thereby improve the accuracy of our estimates.

The first stage of PSM involved estimating a probit regression model of intervention status (SSA exposed vs ineligible) on the set of individual and household level covariates described in the previous section. The predicted probability of intervention status (known as the propensity score) obtained from this regression was then used to match each intervention group woman with control group women. We employed a kernel matching algorithm that calculated a weighted value across the entire control group, with weights that were inversely proportional to the distance (ie, difference in propensity score) of each control observation from the intervention observation. We only considered observations in the two groups that had overlapping propensity scores (known as common support). The average difference in an IPV outcome between the intervention group and the matched control group is defined as the ‘average treatment effect on the treated’ and it can be attributed to SSA under certain conditions.^57^,^63^

### Sensitivity analysis, matching quality tests and subsample analysis

We tested the robustness of our analysis in four ways. First, we considered variations in our PSM algorithm. In addition to Kernel matching, we evaluated matching each intervention group observation with the nearest neighbour (ie, with closest propensity score) in the control group, and matching with three nearest neighbours in the control group. Matching was done with replacement and only among observations that satisfied the common support assumption.

Second, we examined if our PSM results depended on the choice of study period. Instead of evaluating the outcomes of women who were 11–18 years old in 2001 (4 years above and 4 years below the eligibility age of SSA), we considered two shorter study periods: 2 years above and below SSA implementation (13–14 year old intervention and 15–16 years old control group, based on age in 2001) and 3 years above and below SSA (12–14 years old intervention and 15–17 years old control group in 2001). We also considered a longer study period of 5 years above and below SSA (10–14 years old intervention and 15–19 years old control group in 2001).

Third, we considered the possibility that women with higher levels of schooling than others may partner with men that have similarly high levels of schooling (known as ‘assortative mating’). These women may also be situated in households that are richer and have more educated household heads than others. As a result, the risk of them experiencing IPV may be lower. Because the standard of living and the schooling levels of the husband or the household head may be correlated with the intervention (SSA eligibility), we conducted additional PSM analysis that excluded one or more of these indicators from the covariates of the first stage probit regression. If the results remained unchanged, we could argue that there was no or a negligible level of correlation.

Finally, we employed an additional methodology—a state fixed-effects linear probability regression model—to examine the associations of SSA with IPV outcomes. The covariates of this regression were the same as those in the propensity score estimation equation, with the addition of state indicators and a binary indicator of intervention status. SEs were clustered at the state level.

We also tested the quality of matching, that is, whether PSM successfully reduced differences in the background characteristics of the intervention and control groups, through three ways. First, for each covariate of the propensity score estimation regression, we calculated the standardised percentage bias, defined as the difference in mean values between the intervention and control groups divided by the square root of the group difference variance.^64^ A successful PSM should substantially reduce the percentage bias from before to after matching. Second, we re-estimated the propensity score equation using the matched intervention and control samples. For PSM to be valid, the pseudo-R^2^ statistic from this matched-sample regression should be substantially smaller than the pseudo-R^2^ in the original propensity score model. Third, we conducted a likelihood ratio test of the joint significance of the propensity score equation using the matched intervention and control sample. Failure to reject the null hypothesis of joint significance would indicate that after matching, the covariates could not adequately explain the variation in intervention status, that is, PSM was able to successfully reduce systematic differences between the intervention and control groups.

In addition to estimating the associations of SSA with IPV outcomes in the sample of all available women, we separately repeated the analysis for the following subsamples—rural, urban, household where the head had no schooling, general (privileged caste), SC or ST, OBC and those belonging to the two poorest wealth quintiles and two richest wealth quintiles.

### Patient and public involvement

It was not appropriate or possible to involve patients or the public in the design, or conduct, or reporting, or dissemination plans of our research.

## Results

### Summary statistics

Table 1 presents the summary statistics of the study sample. There were 23 679 women who were originally 11–18 years old in 2001 and had data on IPV attitudes in NFHS-5. Among them, 50% were in the intervention group while the other half were in the control group. Rates of women justifying or experiencing IPV were lower in the intervention group as compared with the control group, although the differences were not statistically significant. Intervention group women were younger, had 1.2 years of extra schooling years completed and were more likely to be the child, grandchild, or daughter-in-law of the household head as compared with the control group. They also belonged to slightly larger and richer households, and households with more educated heads and male heads at higher rates than women in the control group. There were no significant differences in the indicators for location, religion or caste.

**Table 1:** Summary statistics of the study sample

	Intervention group	Control group	Difference(intervention–control)
Beating is justified if wife:			
Burns food	0.133 (0.339)	0.144 (0.351)	−0.011
Neglects children	0.281 (0.45)	0.299 (0.458)	−0.018
Argues with husband	0.231 (0.421)	0.235 (0.424)	−0.004
Goes out	0.196 (0.397)	0.205 (0.404)	−0.009
Experienced emotional violence (past year)	0.119 (0.324)	0.121 (0.326)	−0.002
Experienced physical violence (past year)	0.244 (0.43)	0.232 (0.422)	0.007
Age	31.131 (1.355)	35.196 (1.339)	−4.065*
Highest schooling grade completed	7.485 (5.349)	6.311 (5.319)	1.174*
Relationship to household head:			
Self	0.066 (0.248)	0.106 (0.307)	−0.04*
Wife	0.567 (0.495)	0.643 (0.479)	−0.075*
Child	0.054 (0.225)	0.039 (0.193)	0.015*
Daughter-in-law	0.286 (0.452)	0.193 (0.395)	0.093*
Grandchild	0.002 (0.045)	0 (0.014)	0.002*
Age of household head	45.748 (14.306)	46.136 (12.547)	−0.387†
Whether household head is female	0.145 (0.352)	0.171 (0.376)	−0.025*
Household head’s years of schooling	6.742 (5.016)	6.596 (5.044)	0.146
Household characteristics:			
Number of members	5.538 (2.4)	5.25 (2.254)	0.288*
Rural household	0.659 (0.474)	0.66 (0.474)	−0.001
Scheduled caste (SC)	0.222 (0.416)	0.212 (0.409)	0.01
Scheduled tribe (ST)	0.091 (0.288)	0.094 (0.292)	−0.003
Other backward classes (OBC)	0.424 (0.494)	0.427 (0.495)	−0.003
Muslim	0.131 (0.337)	0.132 (0.339)	−0.001
Christian	0.022 (0.145)	0.025 (0.157)	−0.004
Sikh	0.02 (0.139)	0.019 (0.135)	0.001
Household standard of living:			
Wealth quintile 1 (poorest)	0.185 (0.388)	0.19 (0.392)	−0.006
Wealth quintile 2	0.192 (0.394)	0.202 (0.402)	−0.01*
Wealth quintile 3	0.198 (0.399)	0.202 (0.401)	−0.004
Wealth quintile 4	0.21 (0.407)	0.208 (0.406)	0.002
Wealth quintile 5 (richest)	0.215 (0.411)	0.198 (0.398)	0.017†
Sample size	11 777	11 902	

Data are from National Family Health Survey of India, 2019–2021. Mean values with deviations in the parenthesis are presented. Intervention group includes ever-married women who were 11–14 years in 2001 (SSA eligible) and control group includes women who were 15–18 years in 2001 (not eligible for SSA). SC, ST and OBC are government of India designated socioeconomically disadvantaged caste groups.

* p<0.01.

† p<0.05

SSA, Sarva Shiksha Abhiyan.

### Estimated associations of SSA and IPV

Table 2 presents the PSM estimates of the associations between SSA and IPV outcomes. In the main analysis (women aged 11–18 years in 2001, Kernel matching), rates of intervention group women who justified beating by husband or partner for burning food were 2.3 percentage points (pp) lower, for neglecting children were 6 pp lower, for arguing were 4.3 pp lower and for going out without informing the husband or partner were 3.2 pp lower than those in the control group. Intervention group women were also 3.7 pp less likely to experience emotional violence in the past year. In comparison with the control group mean values, these estimates are equivalent to 16%, 20%, 18%, 16% and 31% reduction, respectively. These estimates did not change substantially when we varied the study period by considering 2-year, 3-year and 5-year intervals around SSA introduction in 2001. There was no significant difference in experience of physical violence.

**Table 2:** Propensity score matching estimates of the association between SSA and IPV outcomes

	Sample size	Kernel matching	One-to-one nearest neighbour matching	Three nearest neighbours matching
4 years before and after SSA				
Beating is justified if wife:				
Burns food	23 530	−0.023 (0.011)*	−0.033 (0.014)*	−0.026 (0.012)*
Neglects children	23 530	−0.06 (0.014)†	−0.079 (0.019)†	−0.069 (0.016)†
Argues with husband	23 530	−0.043 (0.013)†	−0.063 (0.017)†	−0.041 (0.015)†
Goes out	23 530	−0.032 (0.012)*	−0.037 (0.017)*	−0.037 (0.014)†
Experienced emotional violence (past year)	20 730	−0.037 (0.011)†	−0.052 (0.016)†	−0.041 (0.012)†
Experienced physical violence (past year)	20 730	0.015 (0.015)	−0.004 (0.02)	0.007 (0.017)
Three years before and after SSA				
Beating is justified if wife:				
Burns food	17 753	−0.023 (0.011)*	−0.028 (0.014)*	−0.023 (0.012)*
Neglects children	17 753	−0.06 (0.014)†	−0.07 (0.02)†	−0.07 (0.016)†
Argues with husband	17 753	−0.043 (0.013)†	−0.047 (0.017)†	−0.046 (0.015)†
Goes out	17 753	−0.032 (0.012)*	−0.042 (0.017)*	−0.041 (0.015)†
Experienced emotional violence (past year)	15 710	−0.037 (0.011)†	−0.055 (0.015)†	−0.05 (0.012)†
Experienced physical violence (past year)	15 710	0.015 (0.015)	−0.004 (0.018)	−0.003 (0.017)
Two years before and after SSA				
Beating is justified if wife:				
Burns food	11 329	−0.023 (0.011)*	−0.031 (0.014)*	−0.017 (0.011)
Neglects children	11 329	−0.06 (0.014)†	−0.05 (0.018)†	−0.051 (0.016)†
Argues with husband	11 329	−0.043 (0.013)†	−0.043 (0.017)*	−0.041 (0.015)†
Goes out	11 329	−0.033 (0.012)†	−0.026 (0.017)	−0.021 (0.013)
Experienced emotional violence (past year)	10 007	−0.037 (0.011)†	−0.052 (0.016)†	−0.046 (0.013)†
Experienced physical violence (past year)	10 007	0.016 (0.015)	0.007 (0.019)	0.011 (0.017)
Five years before and after SSA				
Beating is justified if wife:	29 249	−0.023 (0.011)*	−0.016 (0.014)	−0.027 (0.012)*
Burns food	29 249	−0.06 (0.014)†	−0.06 (0.019)†	−0.062 (0.016)†
Neglects children	29 249	−0.043 (0.013)†	−0.041 (0.017)*	−0.037 (0.015)*
Argues with husband	29 249	−0.032 (0.012)*	−0.038 (0.017)*	−0.034 (0.014)*
Goes out	25 598	−0.033 (0.011)†	−0.042 (0.016)†	−0.036 (0.013)†
Experienced emotional violence (past year)	25 598	−0.037 (0.011)†	−0.041 (0.012)†	−0.05 (0.016)†
Experienced physical violence (past year)	25 598	0.015 (0.015)	0.005 (0.017)	−0.001 (0.02)

Data are from National Family Health Survey of India, 2019–2021. ATT estimates of the association between SSA exposure and IPV outcomes are shown. SEs are in the parenthesis. SC, ST and OBC are government of India designated socioeconomically disadvantaged caste groups.

* p<0.05.

† p<0.01.

ATT, average treatment effect on the treated; IPV, intimate partner violence; OBC, other backward class; SC, scheduled caste; SSA, Sarva Shiksha Abhiyan; ST, scheduled tribe.

In the analysis using one-to-one nearest neighbour matching (4 years above and below SSA introduction in 2001), intervention group women justified IPV at 3.3–7.9 pp lower rates and had 5.2 pp lower rate of emotional violence experience than those in the control group. In additional analysis in which we varied the study period, the negative associations between SSA and justification for IPV were similar and ranged from 1.6 to 7.0 pp, while the negative associations for emotional violence experience ranged from 4.2 to 4.9 pp. Experience of physical violence did not differ between the intervention and control group.

When using one-to-three nearest neighbour matching (4 years above and below SSA introduction in 2001), intervention group women justified IPV at 2.6–6.9 pp lower rates and had 4.1 pp lower rate of emotional violence experience than those in the control group. When we varied the study period, negative associations between SSA and justification for IPV were similar and ranged from 1.7 to 7.0 pp, while the negative associations for emotional violence experience ranged from 3.5 to 4.1 pp. There were no statistically significant associations between SSA and the prevalence of physical violence experienced by women.

Results from additional PSM analysis in which we excluded covariates for wealth quintiles and schooling levels of household head and the woman’s husband are presented in table 3. We examined four alternate PSM model specifications that excluded one or more of these variables. The results (4 years above and below SSA introduction in 2001) were similar to the original analysis—negative associations between SSA and justification for IPV ranged from 1 to 7 pp and negative associations for emotional violence experience ranged from 3 to 4 pp. These estimates were similar when we varied the study period, and those additional results are not presented to save space.

**Table 3:** Additional propensity score matching robustness checks (Four years before and after SSA)

	Sample size	Kernel matching	One-to-one nearest neighbour matching	Three nearest neighbours matching
Analysis without controlling for wealth quintile indicators				
Beating is justified if wife:				
Burns food	23 530	−0.024 (0.011)*	−0.034 (0.015)*	−0.03 (0.012)*
Neglects children	23 530	−0.065 (0.014)†	−0.066 (0.021)†	−0.073 (0.016)†
Argues with husband	23 530	−0.045 (0.013)†	−0.052 (0.018)†	−0.055 (0.015)†
Goes out	23 530	−0.034 (0.012)†	−0.044 (0.019)*	−0.034 (0.014)*
Experienced emotional violence (past year)	20 730	−0.037 (0.01)†	−0.037 (0.016)*	−0.03 (0.013)*
Experienced physical violence (past year)	20 730	0.016 (0.014)	0.024 (0.019)	0.02 (0.016)
Analysis without controlling for the education level of head or wealth quintile indicators				
Beating is justified if wife:				
Burns food	23 530	−0.025 (0.011)*	−0.034 (0.018)	−0.026 (0.013)
Neglects children	23 530	−0.067 (0.014)†	−0.075 (0.023)†	−0.079 (0.017)†
Argues with husband	23 530	−0.047 (0.013)†	−0.04 (0.022)	−0.046 (0.016)†
Goes out	23 530	−0.036 (0.012)†	−0.03 (0.022)	−0.042 (0.016)†
Experienced emotional violence (past year)	20 730	−0.039 (0.01)†	−0.02 (0.019)	0.009 (0.017)
Experienced physical violence (past year)	20 730	0.013 (0.014)	0.019 (0.023)	−0.045 (0.013)†
Analysis controlling for husband’s education level but no wealth quintile indicators				
Beating is justified if wife:				
Burns food	23 478	−0.024 (0.011)*	−0.025 (0.014)	−0.022 (0.012)
Neglects children	23 478	−0.065 (0.014)†	−0.059 (0.019)†	−0.065 (0.016)†
Argues with husband	23 478	−0.045 (0.013)†	−0.047 (0.018)†	−0.043 (0.015)†
Goes out	23 478	−0.034 (0.012)†	−0.029 (0.017)	−0.032 (0.014)*
Experienced emotional violence (past year)	20 682	−0.038 (0.011)†	−0.037 (0.015)*	−0.033 (0.012)†
Experienced physical violence (past year)	20 682	0.016 (0.014)	0.023 (0.019)	0.017 (0.016)
Analysis controlling for husband’s education level and wealth quintile indicators				
Beating is justified if wife:				
Burns food	23 478	−0.022 (0.011)*	−0.021 (0.014)	−0.02 (0.012)
Neglects children	23 478	−0.06 (0.014)†	−0.047 (0.019)*	−0.054 (0.016)†
Argues with husband	23 478	−0.041 (0.013)†	−0.032 (0.017)	−0.032 (0.014)*
Goes out	20 682	−0.028 (0.012)*	−0.018 (0.017)	−0.03 (0.014)*
Experienced emotional violence (past year)	23 478	−0.037 (0.011)†	−0.031 (0.015)*	−0.032 (0.012)†
Experienced physical violence (past year)	23 478	0.015 (0.015)	0.01 (0.02)	0.017 (0.017)

Data are from National Family Health Survey of India, 2019–2021. ATT estimates of the association between SSA exposure and IPV outcomes are shown. SEs are in the parenthesis. SC, ST and OBC are government of India designated socioeconomically disadvantaged caste groups.

* p<0.05

† p<0.01

ATT, average treatment effect on the treated; IPV, intimate partner violence; OBC, other backward class; SC, scheduled caste; SSA, Sarva Shiksha Abhiyan; ST, scheduled tribe.

### Matching quality results, regression analysis and subsample analysis

Results from tests of matching quality are presented in table 4. In all three matching algorithms, standardised percentage bias in covariates reduced substantially from before to after PSM. Mean percentage bias (across all covariates) reduced from 18.7 before matching to 1.8 after matching, and pseudo-R^2^ reduced from 0.8 to 0.003. The p value of the χ^2^ test was 0.7 after matching, indicating that the covariates did not adequately explain the intervention status in the matched sample, as expected. Together, these results show that PSM was valid, that is, it substantially reduced differences in background characteristics of the intervention and control groups. The results were similar in additional analyses in which the study period was varied.

**Table 4:** Propensity score matching quality test results

	4 years before and after SSA		3 years before and after SSA		2 years before and after SSA		5 years before and after SSA	
	% bias before matching	% bias after matching	% bias before matching	% bias after matching	% bias before matching	% bias after matching	% bias before matching	% bias after matching
Age	−308.7†	−2.1	−285.1†	−2.3	−234.1†	−2.5	−322.2†	−2.1
Relationship to household head:								
Self	−8.8†	0.5	−7†	0.6	−5.4†	0.8	−10.1†	0.5
Wife	−18†	−1.9	−13.2†	−1.8	−9†	−1.6	−22.2†	−1.9
Child	7.5†	−0.7	5.5†	−0.6	3.6†	−0.3	9.7†	−0.7
Daughter-in-law	21.6†	1.9	16.4†	1.9	11.6†	1.6	25.8†	2
Grandchild	2.8†	0.4	2.6†	0.5	1.6*	1	3.3†	0.4
Age of household head	−5.3†	−0.8	−4.6†	−0.8	−2.6†	−1.1	−6.8†	−0.8
Whether household head is female	−2.2†	1.3	−2†	1.3	−1.4*	1.5	−2.1†	1.3
Household head’s years of schooling	−0.1	0.1	0.8	0	0.5	−0.6	−1.1*	0
Household characteristics:								
Number of members	10.6†	4.8	7.7†	4.8	4.7†	3.8	12.8†	4.9
Rural household	1.5†	1.3	1.1	1.3	0.6	1.4	2.5†	1.3
Scheduled caste (SC)	0.2	0.9	−0.2	0.8	−1.4	0.7	0.5	0.9
Scheduled tribe (ST)	1.1*	−0.2	0.9	−0.2	1.1	0	1*	−0.2
Other backward classes (OBC)	−0.5	−4.4	−0.7	−4.4	−0.8	−4.9	0	−4.4
Muslim	0.9	1.7	1.3*	1.8	1.1	1.8	1.7†	1.7
Christian	0.3	1.9	0.9	1.9	2†	2.1	−0.3	1.9
Sikh	0.3	6.6*	0.6	6.4*	0.3	7.7*	−0.5	6.7*
Household standard of living:								
Wealth quintile 2	−0.9	0.3	−0.8	0.4	−0.1	0.6	−0.4	0.3
Wealth quintile 3	−0.3	1.6	−0.4	1.6	0.5	1.4	−0.5	1.5
Wealth quintile 4	0.2	−2.7	−0.1	−2.7	0.5	−2.7	0	−2.7
Wealth quintile 5 (richest)	0.5	0.9	1.3*	0.8	0.8	0.6	−0.6	0.9
Mean % bias	18.7	1.8	16.8	1.8	13.5	1.8	20.2	1.8
Pseudo R^2^	0.803	0.003	0.739	0.003	0.593	0.003	0.842	0.004
P value of χ^2^ test	0	0.688	0	0.747	0	0.669	0	0.632

Data are from National Family Health Survey of India, 2019–2021. Standardised percentage bias, which measures the difference in the value of the covariate between intervention and control groups, are presented. SC, ST and OBC are government of India designated socioeconomically disadvantaged caste groups.

* p<0.05.

† p<0.01.

SSA, Sarva Shiksha Abhiyan.

Table 5 presents results from state fixed-effects regression analysis. SSA exposure was associated with 2.2 pp lower rates of justifying beating by husband or partner for burning food and 3 pp lower rates of justifying beating for arguing. The intervention was also associated with 1.8 pp lower prevalence rates of emotional violence experience.

**Table 5:** State fixed effects regression results

	Beating justified if wife burns food	Beating justified if wife neglects children	Beating justified if wife argues	Beating justified if wife goes out	Any emotional violence(past year)	Any physical violence(past year)
SSA eligible	−0.022*	−0.015	−0.03†	−0.019	−0.018*	0.002
Age	−0.009	0.002	−0.008	0.003	0.039	0.055
Age squared	0	0	0	0	−0.001	−0.001
Relationship to household head:						
Self	−0.003	−0.008	−0.01	−0.007	−0.02	−0.008
Wife	0.004	0.006	0.012	0	−0.015	0.017
Child	−0.026	−0.035	−0.026	−0.051*	0.061*	0.059
Daughter-in-law	−0.014	−0.021	−0.017	−0.03	−0.037	0.004
Grandchild	−0.107	−0.088	−0.192†	−0.153†	0.122	0.281
Age of household head	0	0	0	0.001	0	−0.001*
Whether household head is female	−0.001	0.006	−0.003	0.002	−0.002†	−0.006†
Household head’s years of schooling	−0.001	−0.002*	−0.002†	−0.002*	0.001	−0.013
Household characteristics:						
Number of members	0	0.001	−0.001	0	−0.002	0.001
Rural household	0.021†	0.023	0.021*	0.019*	0.002	−0.013
Scheduled caste (SC)	0.018*	0.013	0.007	0.013	0.037†	0.044†
Scheduled tribe (ST)	−0.008	−0.016	−0.008	−0.013	0.006	0.028
Other backward classes (OBC)	−0.002	−0.013	−0.005	−0.004	0.009	0.016
Muslim	0.023*	0.011	0.031*	0.023	0.004	−0.007
Christian	0.004	0.006	−0.011	−0.017	−0.007	−0.002
Sikh	−0.03†	0.02	−0.02	0.017	−0.011	−0.016
Household standard of living:						
Wealth quintile 2	−0.018*	−0.038†	−0.034†	−0.02*	−0.009	−0.013
Wealth quintile 3	−0.028†	−0.04†	−0.063†	−0.042†	−0.018*	−0.04†
Wealth quintile 4	−0.041†	−0.06†	−0.075†	−0.046†	−0.029*	−0.067†
Wealth quintile 5 (richest)	−0.06†	−0.101†	−0.117†	−0.077†	−0.035†	−0.095†
Constant term	0.366	0.314	0.503	0.232	−0.401	−0.543

Data are from National Family Health Survey of India, 2019–2021. State fixed effects are included in all regression models. SEs are clustered at the state level. SC, ST and OBC are government of India designated socioeconomically disadvantaged caste groups.

* p<0.05.

† p<0.01.

SSA, Sarva Shiksha Abhiyan.

Finally, results from subsample analyses are presented in online supplemental table S1. Although the estimates of SSA associations were negative in all subsamples, they were not always statistically significant, likely due to small sample sizes. For IPV attitudes outcomes, significant negative associations were consistently observed in women from urban areas, and in a few cases, among those from rural areas, OBC caste group, and richer wealth quintiles. Negative associations of SSA and experience of emotional violence were observed in most subsamples. For physical violence experience, associations of SSA were statistically insignificant, except for the urban subsample which exhibited a positive relationship.

## Discussion

In this study, we examined a key social benefit of primary education beyond its potential economic effect. Using data from NFHS-5 and employing quasi-experimental matching methods, we find that women who originally benefitted from SSA were 16%–31% less likely to experience emotional IPV or justify IPV than those who were not eligible for the programme. The estimated negative associations with SSA were robust to variations in study period and methodology. They were also larger in magnitude among urban women as compared with rural women. The lower rates of IPV justification and emotional violence among the intervention group suggest that the SSA programme may have contributed to changing attitudes towards IPV and reducing emotional abuse. Education can empower women, increase their awareness of their rights, and improve their ability to negotiate within relationships, which may explain these findings. More research is needed to explore this hypothesis further.

Our findings align with previous studies of schooling and IPV in LMICs, many of which have used regression discontinuity analysis to draw causal conclusions. Two India-based, yet unpublished, studies have used the NFHS 2015–2016 to show that the 1994 regional DPEP programme may have reduced physical, emotional and sexual IPV by up to 26%.^43 44^ In comparison, we used NFHS-5 and evaluated the 2001 national SSA programme to find long-term associations with IPV rates and attitude towards IPV that were similar in sign and magnitude. Among other notable LMIC studies, an evaluation associated tuition-free secondary education policies in 29 sub-Saharan countries with a 5.3 pp reduction in the proportion of women justifying IPV.^65^ A study of a 1997 schooling reform in Turkey that mandated 8 years of primary education for children found that the policy reduced physical violence against rural women, but there was no effect on psychological violence.^24^ However, another study argued that while the policy improved schooling and labour market outcomes of women, it may have increased women’s experience of psychological violence and controlling behaviour perpetrated by partners.^26^ Behrman *et al* linked 1 year of extra schooling grade attained due to the Universal Primary Education policy in the mid-1990s in Uganda with a 9 pp reduction in sexual violence experience by women, but found no effect of a similar policy in Malawi.^25^ Finally, two studies from Peru and China similarly estimated that compulsory schooling laws in the 1980s and 1990s may have improved schooling outcomes and reduced the rates of physical, sexual and psychological violence experienced by women.^28 29^ The authors associated one extra year of schooling due to these policies with 1 pp reduction (equivalent to 17% decrease) in IPV rates in Peru and 3–7 pp reduction in China.

In alignment with the evidence highlighted above, our findings have important policy implications. The United Nation’s Sustainable Development Goal (SDG) 5 calls for gender equality and empowerment of all women and girls, with an SDG 5.2 subgoal target of eliminating all violence against and exploitation of women and girls. However, countries in sub-Saharan Africa and South Asia continue to have among the highest rates of violence against women and girls (VAWGs).^1 2^ There is a robust body of evidence on VAWG prevention interventions, including communication and group training, cash transfers, economic empowerment of women such as skill-building initiatives and programmes that work with boys and men to change gender attitudes.^15^ The SSA programme was associated with reduced levels of justification of IPV and emotional violence, highlighting the importance of educational interventions in addressing gender-based violence. It bolsters the imperative to meet SDG 4 targets of inclusive and equitable quality education for all, contributing in turn to progress in other SDGs.

However, universal primary education alone cannot solve the challenge of reducing IPV or VAWG that are pervasive, persistent and pernicious. As our findings show, national programmes such as SSA may reduce the problem of IPV only to a certain extent, potentially changing women’s perceptions of IPV and reducing cases of emotional violence. There was no significant association with physical violence—which was twice as prevalent as emotional violence in our data—indicating the need for more targeted programmes that can empower women and change the gender attitudes of men. For example, SSA’s predecessor DPEP, which was implemented only in a subset of Indian districts with low female literacy rates and specifically aimed to reduce gender and socioeconomic inequalities in schooling enrolment and retention, was more successful in reducing other forms of IPV in addition to emotional violence.^43 44^

The relatively muted associations of SSA with physical or sexual violence could also be attributed to its smaller effect on schooling outcomes as compared with DPEP or other targeted education programmes globally. DPEP beneficiaries gained up to 1 year of extra schooling in the long term, as compared with 0.2 years of schooling gain for SSA beneficiaries.^32 43 44^ This is likely because DPEP was implemented 7 years prior to SSA when baseline schooling enrolment levels were lower nationally, and more so in the DPEP target districts. The level of education at which a programme is implemented may have additional implications for its social benefits. For example, free and compulsory secondary schooling in 29 countries in sub-Saharan Africa has been linked with 1.6 extra years of schooling gained by women and improvements in their attitude towards IPV, while no effect was seen on IPV outcomes due to free primary schooling policies.^43 44 66^

Regressive gender norms and a preference for sons over daughters remain key drivers of gender inequality in India. Several studies using household survey and administrative data have shown that the dowry system continues to be a key contributor to IPV experience by married women in India.^19^,^2167 68^ Despite being outlawed in 1961 and 1984, dowries are given by the bride’s family to the groom’s family in more than 80% of marriages in South Asia, especially in case of hypergamous marriages where the groom’s family is considered to have a higher socioeconomic status.^67 69 70^ The large dowry payments, both cash and in-kind, often impose severe economic stress on women’s natal households, requiring many years of savings and borrowing from others. Section 498A of the Indian Penal Code of 1983 was among the first to provide protection to women against dowry-related harassment and atrocities. Then, in 2005, the Indian government enacted the Protection of Women from Domestic Violence Act which defined acts of violence against women, specifically providing civil law protection for dowry-related harassment, injury and harm to women from husbands and other relatives. As a result, reported cases of cruelty by husband or relatives increased by an estimated 53% during 2001 to 2018 in India.^68^ However, anti-VAWG laws in India have been criticised both for their lack of clarity and enforcement as well as the potential for misuse.^71^,^73^ Future research in this area should evaluate the effectiveness of schooling expansion and other programmes in reducing harmful practices such as dowries.

While the findings from our analysis are promising, some limitations should be noted. First, we estimated the associations of SSA with IPV using an intention-to-treat approach, without knowing if the eligible beneficiaries actually benefitted from the programme at the individual level. While this approach is commonly used in impact evaluation, there remains a small risk of misclassification of intervention and control observations due to early or delayed school entry or grade repetition. However, previous research on school enrolment patterns in India shows that such measurement errors and associated risk of bias may be small.^74 75^

Second, IPV data were self-reported by women, and they may suffer from under-reporting. Previous studies that used specialised tools for collecting sensitive data, for example, list experiments, have shown that household surveys such as NFHS may underestimate the prevalence of IPV.^76^,^78^ Furthermore, the cross-sectional nature of NFHS data does not allow us to estimate high-quality causal inference effects of SSA. Future research could use longitudinal or preintervention and postintervention data and evaluate the determinants of socioeconomic determinants of SSA rollout to draw causal conclusions.

Third, we considered two cohorts of women just above and below the cut-off age for moving from primary to secondary schooling in our analysis. We also considered a large set of socioeconomic characteristics and employed matching methods that further reduced differences between the intervention and control groups. However, our methods may not fully account for unobserved characteristics that could vary across the two groups and potentially affect our findings. For example, more inherently ambitious women may do better in school and have more equitable gender attitudes, which is not captured in our data.

Finally, due to lack of data, we could not capture variations in the pace and quality of SSA implementation across regions. This is especially important considering the joint role of the central and state governments for education service delivery in India. More analysis with larger sample sizes is required for understanding the potential role played by SSA in reducing IPV across and within states of India.

Despite these limitations, our study shows that at-scale schooling expansion programmes may provide substantial social benefits in terms of reducing VAWGs in India or other LMICs. Considering the complex interplay between gender norms, socioeconomic barriers to girls’ schooling, and intrahousehold bargaining power of women in India, future research should focus on the effectiveness of targeted policies and programmes that can directly address the underlying causes of IPV such as the dowry system, men’s gender attitudes and female economic empowerment.

## Supplementary material

10.1136/bmjph-2024-001530 online supplemental file 1

## Data Availability

Data may be obtained from a third party and are not publicly available.
