# Supplementary material for: Schooling and intimate partner violence: retrospective analysis of India’s Sarva Shiksha Abhiyan using quasi-experimental techniques
Source: BMJ Public Health. 2025 Jul 15;3(2):e001530. doi: 10.1136/bmjph-2024-001530 (PMC12273177; doi:10.1136/bmjph-2024-001530)
Supplement: online supplemental file 1 [file bmjph-3-2-s001.docx]

**Supplementary Appendix**

Table S1: Subsample analysis – propensity score matching estimates of the associations between SSA and IPV

|  | Beating justified if wife burns food | Beating justified if wife neglects children | Beating justified if wife argues | Beating justified if wife goes out | Any emotional violence  (past year) | Any physical violence  (past year) |
| --- | --- | --- | --- | --- | --- | --- |
| Rural | -0.015 (0.013) | -0.046 (0.017)** | -0.037 (0.016)* | -0.024 (0.015) | -0.032 (0.013)* | 0.014 (0.017) |
| Urban | -0.046 (0.017)** | -0.095 (0.025)** | -0.052 (0.022)* | -0.056 (0.022)* | -0.043 (0.018)* | 0.035 (0.026) |
| Household head with no schooling | -0.01 (0.024) | -0.056 (0.031) | -0.041 (0.03) | -0.029 (0.027) | -0.047 (0.023)* | -0.009 (0.031) |
| General caste (privileged) | -0.031 (0.046) | -0.061 (0.061) | 0.002 (0.056) | -0.005 (0.054) | -0.005 (0.023) | -0.001 (0.028) |
| Scheduled Caste (SC) or Tribe (ST) | -0.029 (0.018) | -0.044 (0.023) | -0.045 (0.022)* | -0.019 (0.021) | -0.039 (0.018)* | 0.052 (0.025)* |
| Other Backward Classes (OBC) | -0.015 (0.017) | -0.047 (0.023)* | -0.04 (0.021) | -0.029 (0.02) | -0.061 (0.017)** | -0.001 (0.023) |
| Wealth quintiles 1 and 2 | -0.021 (0.018) | -0.022 (0.022) | -0.034 (0.022) | -0.003 (0.02) | -0.048 (0.017)** | 0.039 (0.024) |
| Wealth quintiles 4 and 5 | -0.026 (0.018) | -0.086 (0.024)** | -0.036 (0.022) | -0.044 (0.022)* | -0.034 (0.017)* | -0.009 (0.022) |

Note: Data are from National Family Health Survey of India, 2019-2021 (NFHS-5). ATT estimates (Kernel matching) of the association between SSA exposure and IPV outcomes are shown. Standard errors are in the parenthesis. SC, ST, and OBC are government of India designated socioeconomically disadvantaged caste groups. * p<0.05, ** p<0.01
